# CT measures of femoral and tibial version and rotational position of femoral and tibial components of knee replacements: limitations in reliability and suitability for routine clinical practice

**DOI:** 10.1007/s00330-021-08483-8

**Published:** 2022-02-10

**Authors:** Andoni P. Toms, Tamam Rifai, Celia Whitehouse, Iain McNamara

**Affiliations:** 1 Department of Radiology, Norfolk & Norwich University Hospital, Colney Lane, Norwich, NR4 7UY UK; 2 Norwich Medical School, University of East Anglia, Norwich, NR4 7TJ UK; 3 Radiology Academy, Colney Lane, Norwich, NR4 7UB UK; 4 Department of Trauma & Orthopaedics, Norfolk & Norwich University Hospital, Colney Lane, Norwich, NR4 7UY UK

**Keywords:** CT, Knee, Arthroplasty, Rotation, Reproducibility of results

## Abstract

**Objectives:**

Rotational malalignment of knee replacements as measured on CT is understood to be associated with poor outcomes. The aim of this study is to measure the inter-rater and intra-rater reliability of measures of femoral and tibial version in the native arthritic knee and postoperative TKR component position using CT.

**Methods:**

Eighty patients underwent CT of the knee before and after total knee replacement. Preoperative femoral and tibial version and component rotation were independently measured by two musculoskeletal radiologists.

**Results:**

Mean differences between and within raters were small (< 1.6°). Maximum 95% limits of agreement for inter-rater and intra-rater comparisons were 8.1° and 7.6° for preoperative femoral version, 9.0° and 7.9° for postoperative femoral rotation, 26.0° and 20.5° for preoperative tibial version, and 24.9° and 23.6° for postoperative tibial rotation respectively. Postoperative ICCs varied from 0.68 to 0.81 (lower 95% CI:0.55–0.72) for both intra- and inter-rater comparisons. Preoperative ICCs were lower: 0.55–0.75 (lower 95% CI:0.40–0.65).

**Conclusion:**

The lower 95% confidence level for ICC of version and rotational measurements using the Berger protocol of TKRs on CT are all less than 0.73 and that the normal range of differences between observers is up to 9° for the femoral component and 26° for the tibial component. This suggests that CT measurements derived from the Berger protocol may not be consistent enough for clinical practice.

**Key Points:**

• *CT is commonly used to measure the rotational profile of knee replacements in symptomatic patients using the Berger protocol.*

• *The limits of agreement for both femoral and tibial component rotation are wide even for experienced observers.*

• *CT measurements of the rotation of knee arthroplasty are not reliable enough for routine clinical use.*

## Introduction

Up to 20% of patients have continued pain or report dissatisfaction after primary total knee replacement (TKR) [1, 2]. Malrotation of either the femoral or tibial components is one possible cause of this dissatisfaction [3]. Problems that have been specifically associated with malrotation of the components are patella maltracking [4, 5], anterior knee pain [6], accelerated wear of the polyethylene liner [7], limitations in the range of motion [8, 9], and abnormal axial rotation motion [10].

The rotational position of the femoral and tibial components is most commonly assessed with computed tomography (CT). While there are a number of methods for analysing the component rotation, the Berger protocol is the most widely used [11–15]. The Berger protocol has been reported to have good inter-rater and intra-rater reliability by some authors [11, 16, 17] but others disagree arguing that the inter-rater reliability is unsatisfactory [18].

The reported statistical methods for calculating the degree of reliability of a measurement vary. The most commonly used measurement is the intraclass correlation coefficient (ICC) as a measurement of inter-rater and intra-rater consistency but caution needs to be applied in interpreting the ICC because not all authors state which type of ICC has been used, including 95% confidence intervals or justify the interpretation of the result [19, 20]. It is recognised that ICC can lead to an overestimate in the degree of reliability and, on its own, is not considered to be an adequate statistical measurement. Tests of reliability should also include Bland–Altman plots (Tukey mean-difference) and 95% levels of agreement [21–23].

The aim of this study was to measure the inter-rater and intra-rater reliability of manual measurements of femoral and tibial versions in the native arthritic knee and postoperative TKR component position using CT.

## Materials and methods

This was a prospective reliability study using data from the CAPAbility trial which is a randomised controlled trial of the JOURNEY II BCS versus GENESIS II TKRs for the treatment of patients with primary osteoarthritis. The study protocol was approved by the East of England – Cambridge Central Research Ethics Committee (reference 16/EE/0230) prior to the start of the trial. The trial is registered on the International Standard Randomised Controlled Trials Number (ISRCTN) registry (reference ISRCTN32315753). Approval was granted by the Health Research Authority (HRA) and Confirmation of Capacity and Capability to conduct the trial has been provided by the Norfolk and Norwich University Hospital Research and Development Office. Exclusion criteria included contralateral knee replacement within 6 months of the primary procedure, fixed-flexion deformity of 15°, patients requiring excessive resection of the distal femur, uncorrectable varus or valgus deformity of ≥ 15°, inflammatory arthritis, previous septic arthritis, previous surgery to the collateral ligaments of the affected knee, and a contralateral TKR that has been implanted less than 1 year previously [24].

For entry into this reliability study, patients had to have undergone both preoperative and postoperative CT of the affected knee. The only exclusion criterion to this study was that the CT was not considered to be of adequate quality to be able to identify the relevant landmarks. The sample size calculation for the CAPAbility study was based on outcomes measured using the Oxford Knee Score. All patients from the CAPAbility study were included in this convenience sample for the reliability study.

### Surgical procedure

The two knee replacements used in this study were the GENESIS II system and the JOURNEY II BCS, both manufactured by Smith and Nephew. The JOURNEY II BCS has been developed to provide improved kinematic outcomes compared to the GENESIS II [25]. Both devices are CE marked and were used within the indication.

The surgical procedure followed the standard surgical approach and technique through a medial parapatellar approach. Both the proximal tibial cut and distal femoral cut were made using an intramedullary cutting guide. Femoral implant sizing was established using the femoral sizing jig before undertaking anterior, posterior, and chamfer cuts. A posterior stabilised component was used in every case. The tibial surface was prepared using the trial template and keel instruments. Posterior femoral osteophytes were routinely removed. All patellae were resurfaced using a 3-peg all-polyethylene patella component. The knee was tested for stability through the full range of motion before the definitive components were cemented in situ*.*

### Computed tomography

Each patient had pre- and post-operative CT examinations (SomatomⓇ Definition AS with 64 detector rows, Siemens) acquiring images through the knee to include the femoral epicondyles and the tibial tuberosity using a standard protocol (FOV: 50 × 60.2 cm, mA: 80, kVp: 120) with reconstructed 1.5-mm axial slices on bone and soft tissue algorithms without specific metal artifact reduction protocols. Patients were imaged supine with their feet loosely bound and held in a comfortable anterior orientation to reduce movement artifact rather than to standardize position.

### Outcome measurements

**Fig. 1:**
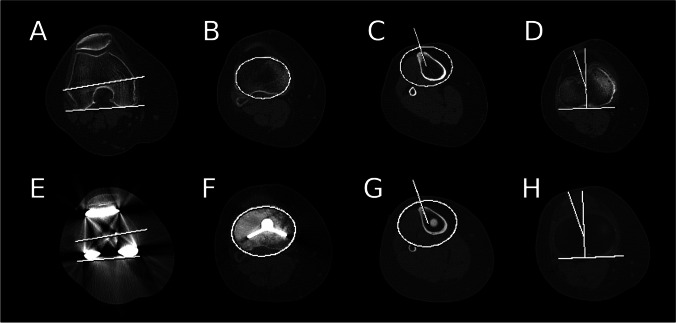
Screen capture of axial CT images including electronic calipers and goniometers demonstrating the method of measurement of preoperative femoral (**A**) and tibial version (**B**–**D**), and postoperative femoral (**E**) and tibial TKR component rotation (**F**–**H**)

Distal femoral and proximal tibial version was measured on the preoperative CT and rotational position of the components of the TKR were measured on the postoperative CT studies. The post-operative measurements were performed according to the Berger protocol [4]. Femoral rotation was defined as the angle subtended by a line drawn between the apices of the femoral epicondyles and a line drawn between the most posterior point of the condylar components of the femoral prosthesis (Fig. 1E). The preoperative measurement of the femoral version used the posterior condylar line as a substitute (Fig. 1A). The rotational position of the tibial component was obtained using the following series of steps. An oval was fitted to the proximal tibial plateau just distal to the cement mantle of the tibial component (Fig. 1H) to find the central axis of the tibia. This oval was copied and posted onto the axial image through the apex of the tibial tuberosity where a line was drawn from the apex of the tibial tuberosity to the centre of the oval (Fig. 1G). This line was copied and pasted to an axial section through the polyethylene line. A second line was drawn as perpendicular to a line drawn along the posterior margin of the polyethylene component (Fig. 1H). The rotational position of the tibial component was taken as the angle subtended by these two lines. The equivalent preoperative measurement of the tibial version (Fig. 1B–D) substituted a line drawn along the posterior tibial plateau for that drawn along the posterior margin of the polyethylene component.

All measurements were performed independently by two musculoskeletal radiologists (6 and 24 years’ experience) both of whom perform rotational TKR profile measurement as part of their routine clinical practice. All measurements were made using dedicated software that allowed superimposition of axial slices and a selection of electronic goniometers on a diagnostic workstation (AW Volumeshare 5, GE Healthcare). Internal rotation of the prosthesis was recorded as a positive angle, external rotation as negative. The observers were free to choose which axial slices they used for their measurements. There was a minimum 4-week interval between the first and second set of observations.

### Statistical analysis

Descriptive statistics along with the Shapiro-Wilks test were used to assess for the normal distribution of data. Inter-rater and intra-rater reliability was assessed using Bland–Altman plots and absolute intra-class correlation coefficients (ICC). All statistical calculations were performed in R (with Psych package for ICC) [26].

## Results

### Descriptive statistics

All data was normally distributed. The mean preoperative angle of the femoral version ranged from 5.14 to 5.60° (95% CI: 4.67, 6.04) and the postoperative femoral component rotation angle from 4.08 to 4.69° (95% CI: 3.52, 5.36). The mean preoperative angle of the tibial version ranged from 19.68 to 21.25° (95% CI: 18.22, 22.54) and the postoperative tibial component rotation angle from 20.49 to 21.89° (95% CI: 18.92, 23.39) (Table 1).

**Table 1:** Descriptive statistics for femoral and tibial version measurements derived from CT. Measures are given for the first and second set of observations for each of the two raters (A and B)

	First observation		Second observation	
Version	Mean angle (SD)	95% CI mean	Mean angle (SD)	95% CI mean
Pre-op femoral A	5.51 (2.01)	5.06, 5.96	5.60 (1.97)	5.16, 6.04
Pre-op femoral B	5.33 (2.06)	4.84, 5.76	5.14 (2.12)	4.67, 5.61
Pre-op tibial A	19.85 (6.36)	18.43, 21.27	19.68 (6.54)	18.22, 21.13
Pre-op tibial B	21.05 (6.71)	19.56, 22.54	21.25 (5.60)	20.00, 22.50
Post-op femoral A	4.16 (2.72)	3.56, 4.77	4.69 (3.04)	4.01, 5.36
Post-op femoral B	4.08 (2.50)	3.52, 4.63	4.33 (2.69)	3.73, 4.92
Post-op tibial A	20.77 (7.34)	19.14, 22.41	21.23 (7.18)	19.60, 22.82
Post-op tibial B	21.89 (6.76)	20.38, 23.39	20.49 (7.05)	18.92, 22.06

### Limits of agreement

The mean differences for all inter and intra-rater measurements were small with only four measurements out of 16 being greater than 1° (Tables 2 and 3). The maximum width of the 95% limits of agreement for inter-rater comparisons for both sets of observations was 8.1° for preoperative femoral version, 9.0° for postoperative femoral component rotation, 26.0° for pre-operative tibial version, and 24.9° for postoperative tibial rotation (Table 2). The maximum width of the 95% limits of intra-rater agreement was a little smaller than the inter-rater agreement, measuring 7.6° and 7.9° for preoperative femoral version and postoperative femoral component rotation, and 20.5° and 23.6° for preoperative tibial version and postoperative tibial component rotation respectively (Table 3).

**Table 2:** Inter-rater reliability statistics for two sets of femoral and tibial version and TKR component rotation measurements derived from CT

	First observation		Second observation	
Version / rotation	Mean difference*	ICC†	Mean difference*	ICC†
Pre-op femoral	0.21 (− 3.86, 4.29)	0.65 (0.49, 0.76)	0.46 (− 3.24, 4.16)	0.72 (0.59, 0.80)
Pre-op tibial	− 1.20 (− 14.27, 11.87)	0.64 (0.48, 0.75)	− 1.57 (− 14.23, 11.08)	0.59 (0.41, 0.72)
Post-op femoral	0.09 (− 4.16, 4.33)	0.79 (0.7, 0.86)	0.36 (− 4.14, 4.86)	0.81 (0.72, 0.87)
Post-op tibial	− 1.11 (− 12.76, 10.54)	0.78 (0.68, 0.85)	0.74 (− 10.69, 12.17)	0.80 (0.71, 0.86)

^*^Mean (95% limits of agreement)

^†^ICC (95% confidence intervals)

**Table 3:** Intra-rater reliability statistics of femoral and tibial version and TKR component rotation measurements

	Rater A		Rater B	
Version / rotation	Mean difference*	ICC†	Mean difference*	ICC†
Pre-op femoral	− 0.09 (− 3.82, 3.64)	0.55 (0.40, 0.66)	0.16 (− 3.43, 3.75)	0.62 (0.49, 0.72)
Pre-op tibial	0.18 (− 8.88, 9.23)	0.75 (0.65, 0.82)	− 0.2 (− 10.47, 10.07)	0.64 (0.52, 0.74)
Post-op femoral	− 0.52 (− 4.13, 3.18)	0.77 (0.69, 0.84)	− 0.25 (− 4.30, 3.80)	0.68 (0.57, 0.77)
Post-op tibial	− 0.45 (− 12.12, 11.22)	0.67 (0.55. 0.76)	1.4 (− 8.66, 11.46)	0.71 (0.61, 0.79)

^*^Mean (95% limits of agreement)

^†^ICC (95% confidence intervals)

### Intraclass correlation coefficients

The range of femoral and tibial postoperative ICCs was 0.68 to 0.81 with lower 95% confidence intervals in a range from 0.55 to 0.72 for both intra- and inter-rater comparisons. The post-operative ICCs were consistently higher than the preoperative ICCs which ranged from 0.55 to 0.75 with a range of lower 95% confidence intervals of 0.40 to 0.65. The range of ICCs for preoperative version and postoperative femoral component rotation varied from 0.55 to 0.81 which was similar to the range of ICCs for tibial component rotation which was 0.59 to 0.80. There was little difference in the ICCs for inter-rater and intra-rater reliability: 0.59 to 0.81 and 0.55 to 0.77 respectively (Tables 2 and 3).

## Discussion

This study presents the results of two different statistical approaches to assessing inter-rater reliability for CT measurements of rotation in the pre- and post-operative knee. ICC is a widely quoted measure of inter-rater reliability but presents problems in interpreting clinically useful results. While Bland–Altman plots are arguably a better method for measuring the reproducibility of a test compared to ICC [21], they have not been previously applied to rotational measurements of ICC. This study presents the Bland–Altman 95% limits of agreement but also includes ICC measurements so that the results can be compared with previously published data.

The results of this study demonstrate that the ICC for measurements of post-operative femoral and tibial component rotation is higher than preoperative CT measurements of femoral and tibial version in the native arthritic knee and that the 95% limits of intra- and inter-rater agreement for measurements of tibial component rotation are much wider than for femoral component rotation. The narrow confidence intervals for the mean of the rotational measurements and ICCs suggest that the sample was an adequate representation of the population being measured and suitable for assessing reliability (Tables 1, 2, and 3).

The ICC measurements for tibial rotation are similar to those in previous reports such as that by Konigsberg et al. (intra-rater ICC = 0.809, inter-rater = 0.67) [16] and Amanatullah et al. (intra-rater ICC = 0.81, inter-rater = 0.52) [18], and the ICC for femoral component rotation were substantially better than those published by Konigsberg et al. (intra-rater ICC: 0.61, inter-rater: 0.39) and others summarised in a subsequent systematic review [11]. These have been interpreted as either “moderate” or “good” reliability for femoral component measurements, and “very good” or “excellent” for tibial rotation depending on the table of descriptive categories used by the authors for interpreting the ICC measurements.

The interpretation of ICC as measure of intra- and inter-rater reliability for the rotational position of TKR components has to date been inadequate and has overestimated the reproducibility of these measurements. There are a number of methods of how ICC might be interpreted whereby ranges between 0 and 1 are given written descriptors. For instance, the often-quoted Landis and Koch descriptors for the strength of agreement are as follows: 0–0.2 slight, 0.21–0.4 fair, 0.41–6.0 moderate, 0.61–8.0 substantial, 0.81–1.0 almost perfect agreement [19]. This differs from another popular interpretation published by Cicchetti et al. that suggests that the ranges should be as follows: < 4.0 poor, 0.4–0.59 fair, 0.6–0.74 good, 0.75–1.0 excellent [27]. In both these examples, and others, these descriptors are to some extent arbitrary. They are a way of describing the degree of mathematical linear correlation between two sets of observations. Interpreting an ICC of 0.62 as “good” using Chiccetti’s method does not mean that the measurement will be obtained with enough consistency to be useful in routine clinical practice or in research. It has been suggested that an ICC of at least 0.81 is required for a test to be considered to be reliable enough for routine clinical practice [28]. In this study, only one out of 16 ICC measurements, post-op femoral rotation, reached this threshold (Table 2).

There is also a view that the reliability of clinical studies should be measured from the lower of the 95% CIs [29]. The 95% confidence intervals demonstrate the range where we can be certain that the true value of the ICC lies. The 95% confidence intervals are wider for smaller sample sizes and the lower limit gives us the minimum ICC for a test that we can know with certainty. None of the lower 95% confidence intervals for ICC published in this study would reach the 0.81 thresholds for reliability. This correlates with other authors’ experience [18] and is not considered in a previous systematic review of the data for measuring TKR rotation with CT [11].

**Fig. 2:**
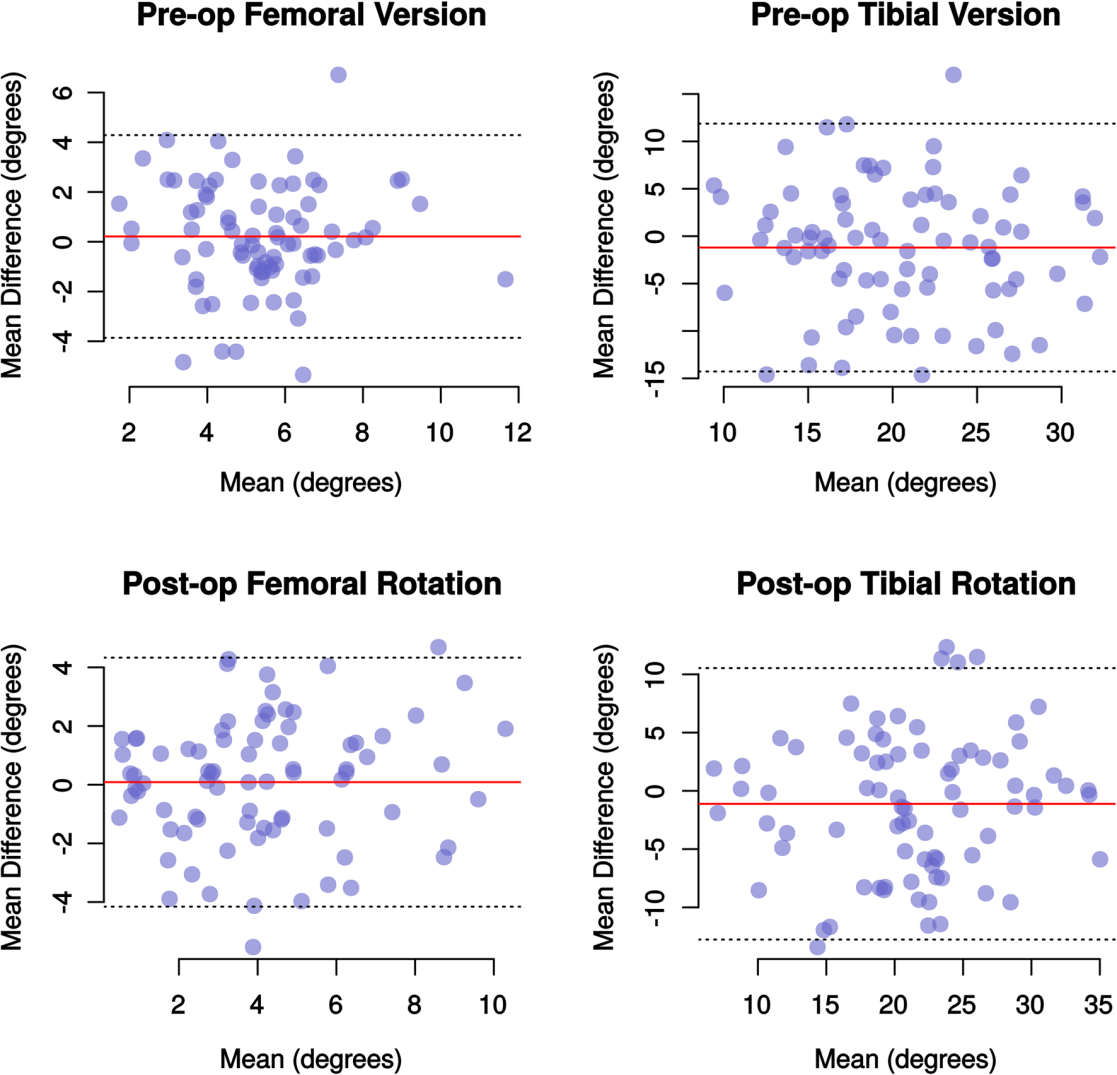
Bland–Altman plots demonstrating the mean difference and the 95% limits of agreement between the two observers for the first set of version and rotational measurements. The width of the limit of agreement is noticeably larger for measures of tibial than for the femoral version and rotation

**Fig. 3:**
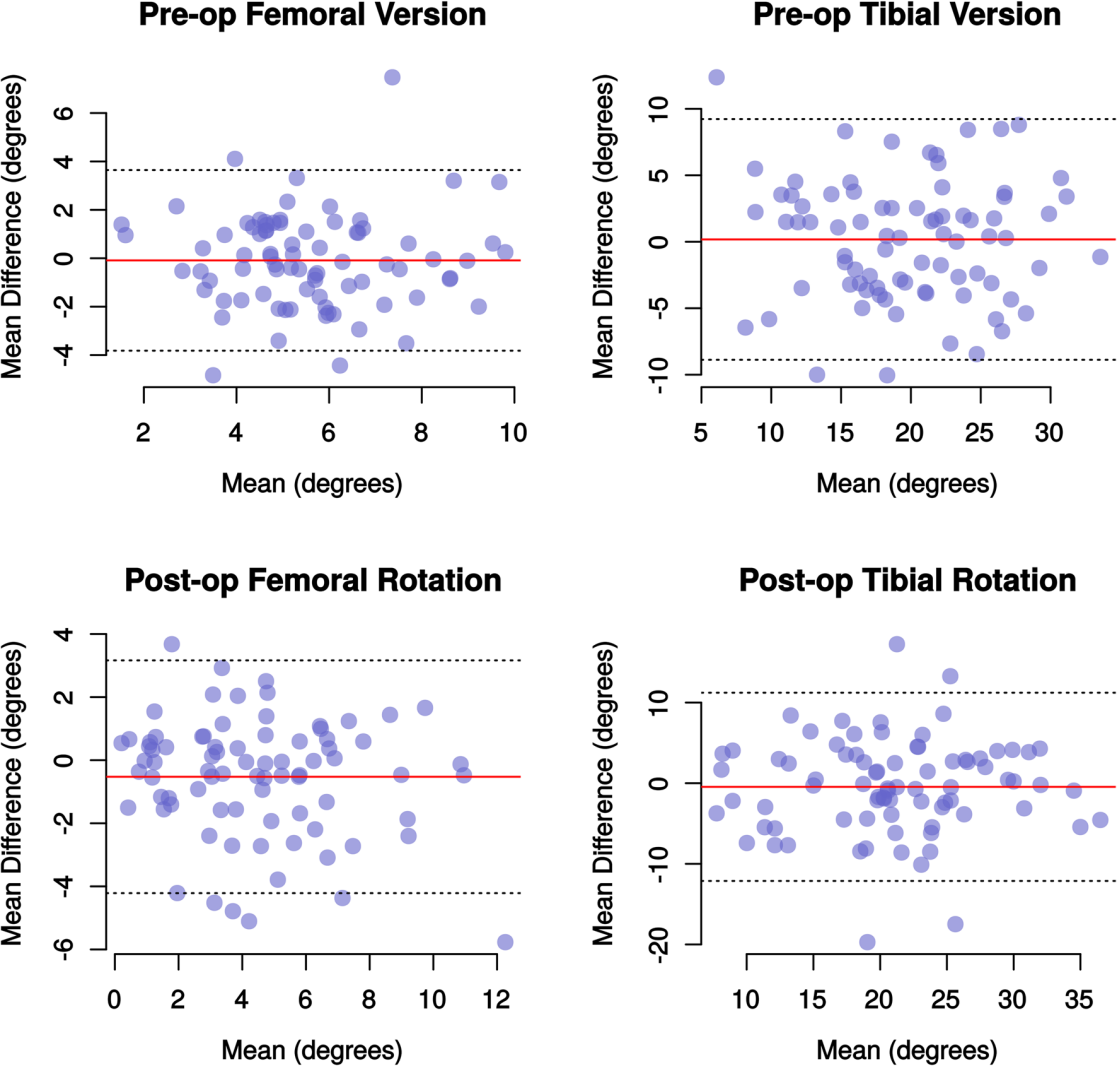
Bland–Altman plots demonstrating the mean difference and limits of agreement between the first and second observations of a single rater. Again, the limits of agreement for tibial measurements are much wider than for femoral measurements

There are other limitations of only using ICC for reliability studies. If repeated measurements differ always by the same amount, then they will be consistent (with a high ICC) but discordant, and the magnitude of the difference between observations may depend on the size of the observations [30]. These effects and the limits of agreement can, and should, be demonstrated with Bland–Altman plots for any reliability study [21] (Figs. 2 and 3). To our knowledge, only one publication reporting the reliability of the rotational position of TKR has reported limits of agreement, and this is in a non-standard form [31].

The Bland–Altman plots from our study demonstrate no funneling which means that size of the differences between observations is not proportionate to the variable being measured. The mean differences for all comparisons were small, less than 1.6°, but the 95% limits of agreement were larger. The 95% limits of agreement varied between 7.3 and 9.1° for femoral measurements and 20.7 and 26.0° for tibial measurements. This means that 95% of all measurements of tibial rotation differed by up to 26° and that it is normal for tibial measurements to vary by this much.

This amount of variation seems at first to be extraordinary but the explanation lies in the description of how each measurement is obtained. For femoral rotation, each rater has to select their optimal axial section (this choice may vary between observations) and draw two lines with an electronic caliper between four anatomical points. Two to three degrees of variation at each step is all that is required for some measurements to vary by up to 9 degrees. Measuring tibial rotation requires the independent selection of three separate axial slices and the setting of four sets of calipers. Two to three degrees error at each of these seven separate steps will account for the outer limit of differences of 26°.

There are other possible sources of error that might compound these results. One interpretation is that the observers are either inexperienced or not competent. Inexperience is unlikely to be a significant factor in this study because both raters are subspecialty trained musculoskeletal radiologists who work closely with the local knee surgeons. They routinely perform TKR rotational profiles measurements in their normal clinical practice and there was no difference in performance between the junior and senior rater. Incompetence is also unlikely to be a factor because the senior rater has a track record in performing imaging reliability studies with narrow limits of agreement in other settings. The conspicuity of bony landmarks can be limited by beam hardening and photopaenic artefacts caused by orthopaedic hardware. Therefore, optimising metal artefact reduction parameters could have an effect on the reliability of the test but interestingly the post-operative ICCs were higher than the preoperative ICCs which suggests that the metal artefact did not have a significantly deleterious effect on the outcome. Previous reports of rotational TKR alignment have not included measurements of the native preoperative femoral and tibial rotation. We included these to determine whether or not CT measurements of preoperative landmarks were reliable enough to use in further studies where the degree of alignment of the TKR compared to the native knee could be correlated with clinical outcomes. One possible source of increased error in the preoperative measurements is the location of the coronal plane of the proximal tibia. The equivalent plane in the postoperative knee is clearly defined as the posterior margin of the polyethylene component but the posterior margin of the proximal tibia is often distorted by marginal osteophytes and therefore a line drawn through the maximal oblique width of the proximal tibial epiphysis may be more suitable.

A number of authors have reported greater ICC than in our study but some of these have used semi-automated methods for their measurements or have not stated whether or not measurements were obtained repeatedly from the same slice or if the raters’ choice of the axial slice was completely independent [31, 32]. These studies are either not or may not be comparable. There are a number of alternative methods to the Berger protocol for assessing femoral and tibial rotation, which may be less susceptible to human measurement error but many of these include a similar number of steps and are therefore likely to be as problematic [12–15]. There is evidence that 3D-CT may be more reliable than measuring from simple axial sections but none of these techniques are as well established as the Berger and other similar protocols [33]. A final question is whether or not the type of prosthesis has an effect on the result. It is possible that the shape and CT attenuation of some prostheses might allow for more reproducible results in which case the results from this study may not be generalisable. Although two different types of the prosthesis were included in this study, subset analysis has not been performed.

In conclusion, the results of this study show that the lower 95% confidence level for ICC of rotational measurements of the femoral and tibial components of total knee replacements using the Berger protocol of TKRs on CT are all less than 0.73 and that the normal range of differences between observers is up to 9° for the femoral component and 26° for the tibial component. These limitations in reliability suggest that this technique may not be suitable for routine clinical practice.

## Abbreviations

CT: Computed tomography
ICC: Intraclass correlation coefficient
TKR: Total knee replacement

## Declarations

### Guarantor

The scientific guarantor of this publication is Andoni P. Toms

### Statistics and biometry

One of the authors has significant statistical expertise.

### Study subjects or cohorts overlap

The methodology *only* for the CAPAbility clinical trial has been reported in:

Clarke C, Pomeroy V, Clark A, Creelman G, Hancock N, Horton S, Killett A, Mann C, Payerne E, Toms A, Roberts G, Smith T, Swart AM, McNamara I. CAPAbility: comparison of the JOURNEY II Bi-Cruciate Stabilised and GENESIS II total knee arthroplasty in performance and functional ability: protocol of a randomised controlled trial. Trials. 2020 Feb 24;21(1):222.

No study subjects or cohorts have been previously reported.

### Methodology

• prospective

• cross-sectional study

• performed at one institution
